# Treatment of Chlorine Gas Poisoning

**Published:** 1918

**Authors:** 


***Treatment of Chlorine Gas Poisoning.*** By A. STUART HEBBLETHWAITE, Capt. R. A. M. C. (T. F.). Abs. from the *Jour. R. A. M. C.*, Dec. 1917, Vol. XXIX, N°. 6.

The author recommends the following treatment for slight cases:

1. Ammonia capsules.
2. Atropine 1/50 grain hypodermically.
3. Fluid or light diet for 24 hours.
4. Open air in a marquee during the day to ensure constant change of air.
5. An expectoránt mixture of ammonia carbonate on the second day.
6. Evacuation to base on the 3rd day if condition admits.

After the administration of the ammonia and atropine from 15 to 25 ounces of blood are withdrawn. After 48 hours a light diet may be given, and an expectorant mixture of ammonia carbonate. If venesection is practised on admission, these cases do well. Oxygen administered through a mouthpiece is beneficial.

In cases of heart failure, venesection does no good. Stimulants, such as strychnin, digitalin, and pituitrin have given good results.

In connection with the other treatments, venesection is strikingly effective in relieving cyanosis, congestion in the lungs, and acute headache, besides inducing sleep. Early venesection is most effi cacious. If it is performed late, cardiac stimulants are required.

Ammonia carbonate given on the third day to patients with copious frothy sputum is helpful in clearing up the chest.

Ipecacuanha administered in quantities too small to cause vomiting, cleared the chest up equally well.

Morphia is useful not only for promoting sleep but also in reducing the pleuritic pain at the costal margin caused by the emphysematous condition of the margin of the lung. It may be used when applications of iodine, mustard leaves, and fomentations have failed.

Postural treatment by raising the foot of the bed is helpful in cases so severe that the reflex of coughing is abolished. If a patient is conscious, he should lie on his side with the lower end of the bed raised a foot. An unconscious patient may be placed on his back, and the lower end of the bed raised two feet. Thus the pulmonary secretions are brought down.

Pulmonary emphysema giving rise to agonizing pains begins usually on the third day along the margin of the lung. Painting with strong iodine is effective. Morphia, if it can be administered with safety, affords relief.

